# Uncertainty Quantification of Fatigue Life for Cement-Stabilized Cold Recycled Mixtures Using Probabilistic Programming

**DOI:** 10.3390/ma18194439

**Published:** 2025-09-23

**Authors:** Hao Liu, Jiaolong Ren, Lin Zhang, Qingyi Lv, Shenghan Zhuang, Hongbo Zhao

**Affiliations:** 1 School of Civil Engineering and Geomatics, Shandong University of Technology, Zibo 255000, China; 18154346079@163.com (H.L.); worjl@sdut.edu.cn (J.R.); kennylzh@connect.hku.hk (L.Z.); 23507030868@stumail.sdut.edu.cn (Q.L.); 2 Department of Bridge Engineering, Southwest Jiaotong University, Chengdu 610031, China; shenghanzhuang@my.swjtu.edu.cn

**Keywords:** fatigue, CSCRM, uncertainty quantification, probabilistic programming, PyMC3

## Abstract

The assessment of fatigue life is important for the design of pavement materials because fatigue cracks are one of the most common types of failure in pavement structures. The fatigue test is commonly used to determine the fatigue life. However, there are lots of uncertainties, such as the construction environment and personal operations, during the fatigue test due to the complexity of the pavement materials. Determining the fatigue life of pavement materials under uncertainty is a challenging task. In this study, considering cement-stabilized cold recycled mixtures (CSCRMs) as an example, an uncertainty quantification (UQ) method based on PyMC3, a novel and powerful probabilistic programming package, was developed to address the uncertainty in fatigue behavior based on fatigue tests. Probabilistic programming was employed to characterize the uncertainty of fatigue life based on fatigue test data and the fatigue life formula. The uncertainty of fatigue life was quantified by determining the unknown coefficient of the fatigue life formula. Two independent datasets for the CSCRM were used to illustrate and verify the developed method. The coefficients of determination (R^2^) for the prediction results of fatigue life were higher than 0.96, based on the obtained formula and test data. The maximum and average errors of the coefficients determined using the fatigue equation were less than 11% and 7%, respectively. The verification demonstrates that the predicted fatigue life closely agrees with the test data, and the determined coefficients of the fatigue equation are in excellent agreement with prior findings. The developed method avoided complex statistical computations and references. The UQ can evaluate the fatigue life and its uncertainty and significantly enhance the understanding of the fatigue behavior of the CSCRM.

## 1. Introduction

The assessment of fatigue life is essential to the design of pavement materials because fatigue cracks are one of the most common failure modes in pavement structures [1,2]. However, investigating the fatigue life of road materials poses a challenge for typical engineering organizations due to the complexity of fatigue tests, which require high equipment costs and significant time commitments, and face issues with data interpretation. Meanwhile, fatigue life is very discrete and not deterministic due to the uncertainty, such as materials being inherently discrete, the field environment, etc. Uncertainty is an inherent property of the fatigue behavior of pavement materials. It is critical to quantify and consider uncertainty when determining the fatigue life of pavement materials.

In recent decades, various methods have been proposed to determine the fatigue life of pavement materials. The prediction methods for characterizing fatigue life are mainly divided into two categories: phenomenological-based and mechanical-theory-based [3]. The phenomenological-based fatigue prediction method mainly establishes empirical or semi-empirical formulas through experimental data, and it represents the macroscopic law of fatigue damage. Numerous studies have been conducted in this regard. Azarhoosh et al. [4] predicted the fatigue life of a precipitated calcium carbonate-modified asphalt mixture using nonlinear genetic-based models. Fang et al. [5] and Ren et al. [6] established fatigue life prediction models for rubber asphalt mixtures and semi-flexible composite pavement mixtures based on damage evolution, respectively. Li et al. [7] proposed a method for calculating fatigue life that considers the combined effects of creep damage and fatigue damage. Gajewski et al. [8] calculated the fatigue life of high-modulus asphalt concrete by considering various definitions of fatigue life. Fedrigo et al. [9] characterized the laboratory fatigue behavior of lightly cement-stabilized materials using South African materials and compared it with that of South African and Brazilian materials. Seif et al. [10] investigated the relationship between the fatigue life of asphalt mixtures and asphalt binders using the rate of change in dissipated energy. Seitllari et al. [11] revealed the link between the fatigue life and the sample size of asphalt concrete. Fedrigo et al. [12], Ji et al. [13], and Zhao et al. [14] analyzed the effect of compaction methods and material composition on the fatigue life of cement-stabilized cold recycled mixtures. Xu et al. [15] created a fatigue life prediction model for the fatigue reliability design of steel bridge deck asphalt pavement. Ingrassia et al. [16] predicted pavement fatigue life by using KENPAVE and FLEXPAVE software. Złotowska et al. [17] proposed a method for predicting fatigue life based on the AASHTO 2004 equations and laboratory fatigue testing results of asphalt concrete mixes used in pavement design. Fedrigo et al. [9] evaluated the fatigue life of pavements using a novel Brazilian design method, the *South African Pavement Engineering Manual*, and AASHTO Pavement Mechanistic-Empirical Design software. Omrani et al. [18] even used machine learning models—random forest and XGBoost—to predict the fatigue life of emulsified asphalt cold recycled mixtures, among which XGBoost achieved more accurate predictions of fatigue life. In contrast, the mechanism-based method starts from the intrinsic mechanism of fatigue failure. With the development of viscoelastic continuum damage mechanics (VECD), it has been widely applied in the field of road engineering. Zhang et al. [3] explored the fatigue characteristics of asphalt mixtures using VECD theory and derived a mechanical prediction equation. To further improve accuracy, a temperature adjustment coefficient was also introduced, enabling the prediction error to be controlled within 20%. Yang et al. [19] investigated the effects of different factors within various S-VECD-based fatigue prediction models on fatigue life. Han et al. [20] developed a physics-informed neural network embedded in VECD, namely the PINN-AFP model. This model can accurately predict the damage characteristic curve of asphalt mixtures using a small amount of experimental data. However, uncertainty, which is essential for the fatigue life of pavement materials, was not considered in these studies.

Uncertainty is an essential attribute of engineering materials that influences their performance. The predicted results regarding fatigue life may deviate from reality owing to uncertainty, resulting in unknown engineering risks. Understanding this uncertainty can improve risk resistance and engineering reliability. To address the uncertainty, various probabilistic methods have been developed to assess the fatigue life of pavement materials. Statistical and data-fitting methods have been utilized to predict fatigue life based on fatigue experiments [21,22,23,24,25]. Luo et al. [26] studied the fatigue life of rubberized asphalt concrete using a probabilistic method. A fatigue life model was developed for plain concrete and fiber-reinforced concrete by considering the observed influences of frequency and the stress ratio [27]. Ding et al. [28] proposed a stochastic fatigue damage model based on the physical mechanisms of concrete fatigue. A stochastic damage model was developed to determine the fatigue life by considering the randomness of the material composition [29]. Bressi et al. [30] conducted a comparative assessment of the environmental performance of sixteen types of cement-treated base mixtures. Das et al. [31] also established a damage-based fatigue prediction model, which exhibited good performance under Monte Carlo simulation. Another approach that combines the probabilistic method with VECD has also been proven to effectively account for uncertainties and improve the fatigue life prediction results of asphalt mixtures [32,33,34,35].

During the maintenance and repair of highway engineering, a significant amount of waste engineering materials is produced. Traditional waste disposal methods can lead to environmental pollution and increased costs, so many of these waste materials have been repurposed for use in road construction [36]. Among these, cement-stabilized materials are commonly utilized in road bases. In particular, cement-stabilized cold recycled mixtures (CSCRMs), which consist of cement, cement-based road waste materials (CRWMs), asphalt-based road waste materials (ARWMs), and natural aggregates (NAs), have gained considerable attention due to their cost-effectiveness. Chen et al. [37] studied how different sequences of mixing materials affect the mechanical properties of CSCRMs and the interfacial bonding between recycled aggregates and cement. Li et al. [38] and Ren et al. [39] examined the influence of cement content on the mechanical behavior of CSCRMs and further explained how cement impacts CSCRM performance by analyzing its effect on voids. Khan et al. [40] showed that adding cement improves the strength of CSCRMs and revealed the microscopic mechanisms behind this increase in strength. Hou et al. [41] and Xiang et al. [42] focused on the mechanical properties of CSCRMs when using 100% CRWM content. However, the fatigue performance of CSCRMs has received relatively little attention. Ji et al. [13] and Jiang et al. [43] explored how varying material contents affect the fatigue life of CSCRMs; additionally, Ren et al. [36] applied a data-driven method to predict CSCRM fatigue life. Zhang et al. [44] developed a fatigue life prediction model for CRCSMs by integrating neural networks with an attention mechanism.

Uncertainty quantification (UQ) is a useful tool for evaluating uncertainty from the perspective of probability logic. It combines uncertain and incomplete test data from various sources and provides an uncertainty assessment, which is used to update the accuracy of the model [45]. Consequently, considering UQ during material design and pavement construction significantly improves the reliability and safety of pavement infrastructure. However, the uncertainty of the fatigue test data has not been quantified in existing studies. The uncertainty of the fatigue life is also not considered in material design and pavement construction. In this study, UQ is adopted to characterize the fatigue behavior of cement-stabilized cold recycled mixtures (CSCRMs) based on the laboratory tests.

On the other hand, various UQ methods have been proposed, involving different mathematical and computational theories, and the selection of UQ methods depends on specific engineering problems and uncertainty characteristics. In this study, probabilistic programming is adopted to capture the uncertainty of the fatigue life of the CSCRM. Compared with other UQ methods, probabilistic programming has the following advantages. Firstly, probabilistic programming can check the inner structure of the model and examine the model parameters that have been learned when the model is not a “black box”, which is beneficial for explaining system behavior. Secondly, probabilistic programming can merge domain knowledge into the model. Thirdly, probabilistic programming can flexibly establish a Bayesian model and improve computational iteration efficiency to reduce the requirement of the mathematical level for the user and reduce the time cost during model establishment.

This study aims to develop a novel UQ framework to capture the uncertainty of the fatigue behavior of the CSCRM based on indirect tensile fatigue tests. An empirical formula was used to determine the fatigue life based on a fatigue test. Probabilistic programming was employed to address uncertainty during the fatigue test. The unknown coefficient of the empirical formula was determined using PyMC3, an excellent tool for probabilistic programming. The developed method provides a scientific and reliable way to consider uncertainty when determining the fatigue life of pavement materials. The CSCRM was adopted to illustrate and verify the above method. The CSCRM contains four raw materials. A complex material composition will bring about more significant uncertainty during the fatigue process [46], which is beneficial for explaining the method proposed in this study. The organization of this study is as follows. First, Section 2 introduces the fatigue test and the fatigue life equation to characterize the fatigue behavior. Secondly, in Section 3, the UQ-based fatigue life was developed to consider uncertainty based on the idea and algorithm of UQ, and the detailed procedure of the developed framework is presented. Then, the developed framework is illustrated using the CSCRM. Lastly, summaries and conclusions are drawn from the results. It shows that UQ provides a reasonable approach for capturing fatigue behavior and quantifying the uncertainty of fatigue life.

## 2. The Fatigue Test and the Fatigue Life Equation

### 2.1. Fatigue Laboratory Test

A CSCRM is a type of recycled road material containing cement-based road waste materials (CRWMs), asphalt-based road waste materials (ARWMs), natural aggregates (NAs), and cement, and has been widely studied in recent years due to its good performance–cost ratio [14,47]. The NA (limestone), CRWMs, and ARWMs used in this study were all obtained from the National Highway in Chuzhou, Anhui Province. From the viewpoint of material composition, the CSCRM is similar to cement-stabilized macadam. The major difference between the two mixtures is the aggregate type. The aggregates used in the cement-stabilized macadam are all NAs, and those used in the CSCRM are composed of NAs, CRWMs, and ARWMs. Although their strength characteristics are different due to the different aggregates, a CSCRM with an appropriate composition can replace cement-stabilized macadam as a pavement base material [39].

An indirect tensile fatigue test is conducted to obtain the fatigue life data for the CSCRM based on the Chinese standard (JTG D50, 2017). The CSCRM samples are compacted using the static pressure method under the optimum moisture content. The optimum moisture content is measured using the heavy compaction method. A half-sine stress control mode under the frequency of 15 Hz was adopted based on four types of stress levels (0.5, 0.6, 0.7, and 0.8, calculated using Equation (1)). The technical parameters of the raw materials (CRWMs, ARWMs, NAs, and cement) and the design parameters, including the maximum dry density (MDD), the optimum moisture content (OMC), and the splitting strength (i.e., indirect tensile strength), of the CSCRMs are provided in Appendix A. The detailed process of the fatigue test can be found in previous studies [36,48] and is not covered here. Six samples are successfully tested for each stress level and CSCRM composition in both splitting strength tests and fatigue tests. The fatigue lives of various CSCRMs are listed in Appendix B. The fatigue test results presented in Table A5 are mean values of the six parallel samples for each mixture tested under each stress level. In addition, according to the Chinese experimental standard “Test Methods of Materials Stabilized with Inorganic (JTG E 51-2009)” [49], the results of the fatigue test are valid when the correlation index between the mean values of fatigue life and the stress level is higher than 50%. The data on fatigue life presented in Table A5 are consistent with this law.

(1) $$\sigma =\frac{\sigma_{d}}{\sigma_{s}}$$

where *σ* is the stress level, *σ_d_* is the load applied in the fatigue tests (MPa), and *σ_s_* is the 90d ultimate indirect tensile strength (MPa).

### 2.2. The Fatigue Life Equation

In a previous study [36], a symbolic-regression-based equation was established to predict the fatigue life of the CSCRM, as shown in Equation (2).

(2) $$fatigue\;life=\left\{\begin{array}{c} \frac{a}{x_{1}{\left(x_{0}+x_{1}\right)}^{2}+\left(x_{0}+x_{1}-b\right)\left(x_{0}+x_{1}+c\right)+x_{3}}\begin{array}{cc} & x_{3}\leq d \end{array}\\ \frac{ax_{2}}{x_{1}{\left(x_{0}+x_{1}\right)}^{2}+\left(x_{0}+x_{1}-b\right)\left(x_{0}+x_{1}+c\right)+x_{3}}\begin{array}{cc} & x_{3}>d \end{array} \end{array}\right.$$

where *a*, *b*, *c*, and *d* are the coefficients determined by fatigue test data using Bayesian inference; *x*_0_, *x*_1_, and *x*_2_ are the contents of the CRWMs, ARWMs, and cement; and *x*_3_ is the stress level.

Once the fatigue life equation is obtained, the fatigue properties can be determined based on the relationship between the fatigue life and its influencing factors for the CSCRM. However, uncertainty is inevitable in materials owing to factors such as the complexity of the material composition, the engineering environment, and laboratory tests. In this study, UQ was implemented to capture the fatigue lives and their uncertainty based on Equation (2).

## 3. Uncertainty Quantification for Fatigue Life Analysis

### 3.1. Uncertainty Quantification

The uncertainties of the inputs and parameters are propagated to the outputs of the engineering system, and the resulting system response is uncertain. Uncertainty is an inherent property of the cement material. UQ is used to capture and quantify the uncertainty relationship between the input and output in an engineering system. In general, UQ is used to investigate the propagation of uncertainty in the response of an engineering system, where the uncertainty of the input of the engineering model is based on a physical model. In recent decades, various computational algorithms have been developed for UQ [50,51]. This study regarded probabilistic programming as the UQ method for approaching the fatigue behavior of the CSCRM under uncertainty based on Markov chain Monte Carlo (MCMC) sampling techniques. Probabilistic programming is the automation of Bayesian inference and combines machine learning, statistics, and programming languages. It uses formal semantics, compilers, and other tools to build effective inference evaluation models based on inference algorithms and statistical theory.

Probabilistic programming enables the extraction of unknown information from observed data using physical models. The uncertainty within the system is represented through probabilistic features incorporated into the simulator. Inference algorithms can automatically infer unknown mechanisms and uncertain parameters of an engineering system based on observed data. Over the past decades, several probabilistic programming tools have been developed, including BUGS, Stan, AutoBayes, and PyMC3. Advanced Markov Chain Monte Carlo (MCMC) methods, such as Hamiltonian Monte Carlo and the No-U-Turn Sampler, can handle high-dimensional and complex posterior distributions, allowing the use of sophisticated models without requiring extensive expertise in fitting techniques. In this research, the Python-based probabilistic programming package PyMC3 was employed to assess the fatigue behavior and fatigue life of the CSCRM. PyMC3 is a modern, open-source package featuring an intuitive, readable, and powerful syntax that closely resembles the natural language used by statisticians to define models [52]. It was used here to address general Bayesian prediction and statistical inference challenges.

In this study, UQ was employed to characterize the fatigue behavior of the CSCRM and the associated uncertainty. The coefficient of the fatigue life equation was obtained based on fatigue tests and probabilistic programming. UQ provides a helpful, reasonable, and promising tool for characterizing the fatigue behavior and its uncertainty.

### 3.2. Uncertainty Quantification of Fatigue Life Using PyMC3

To assess uncertainty in the fatigue life of the CSCRM, PyMC3 is employed to estimate the coefficients of the fatigue life equation, the corresponding fatigue life, and their uncertainties using fatigue test data. The predicted fatigue life *fl* is modeled as normally distributed observations, with an expected value *σ_f_* that is a nonlinear function of the unknown uncertain coefficients in the fatigue model, as defined by Equation (2).

(3) $$fl\sim N(\mu_{f},\sigma_{f}^{2})$$

(4) $$\mu_{f}=f(X,C)$$

where *f* denotes the fatigue life, *X* = (*x*_0_, *x*_1_, *x*_2_, *x*_3_) is a vector that denotes the CRWM content (0%, 6.25%, 12.5%, 18.75%, 25%, 37.5%, 50%, 56.25%, 75%, and 100%), ARWM content (0%, 6.25%, 12.5%, 18.75%, 25%, 37.5%, 50%, 56.25%, 75%, and 100%), cement content (4% and 5%), and stress level (0.5, 0.6, 0.7, and 0.8). The detailed data of the fatigue lives and material compositions are provided in Appendix B. *C =* (*a*, *b*, *c*, *d*) is a vector that denotes the coefficients of the fatigue equation (Equation (2)). A uniform distribution [*C_l_*, *C_u_*] is applied to the unknown coefficient of the fatigue equation (Equation (2)). Cl and Cu are the lower and upper bounds of *C*, respectively. In this study, a uniform distribution was used to represent the weak information about the actual unknown coefficient. According to the specific cement material, other distributions, such as the normal distribution, can be used based on the known information.

(5) $$C\sim U(C_{l},C_{u})$$

By using PyMC3 to define the model described above, a posterior estimate of the unknown coefficient in the fatigue equation is calculated according to Equation (2) in the next step. Depending on the problem′s objective and the model′s structure, there are two approaches to estimating the unknown coefficients: one involves using an optimization technique to identify the maximum a posteriori estimate, and the other is to apply MCMC sampling to generate a summary of samples from the posterior distribution.

### 3.3. UQ Procedure 

**Figure 1:**
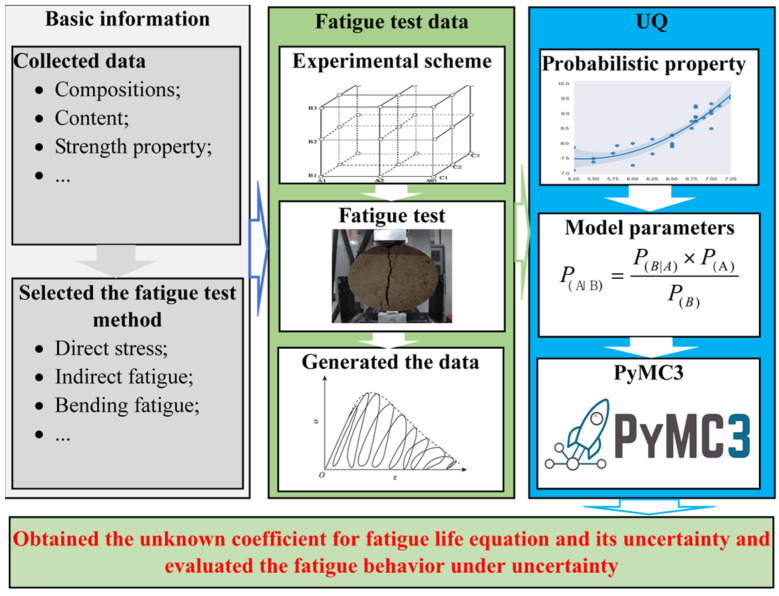
The flowchart of the developed framework.

UQ was utilized to determine the unknown coefficient of the fatigue life equation and evaluate the uncertainty of the fatigue behavior of the CSCRM based on probabilistic programming using PyMC3. An indirect fatigue test was used to construct the dataset, which was used to capture the fatigue behavior based on probabilistic programming. The MCMC was adopted to determine the fatigue life and its uncertainty using PyMC3. The flowchart of the UQ of fatigue life is shown in Figure 1. The detailed UQ procedure for fatigue behavior is as follows:

Step 1: The fatigue test method is selected, and the experimental scheme is determined based on the experimental design.

Step 2: The fatigue test is conducted, and the data are generated.

Step 3: The prior and posterior information and their probabilistic properties are determined.

Step 4: The unknown lower and upper limits of the unknown coefficient and other parameters of PyMC3 are determined.

Step 5: Probabilistic reasoning based on the MCMC is implemented using PyMC3.

Step 6: The unknown coefficient for the fatigue life equation and its uncertainty are determined, and the evaluation of fatigue behavior under uncertainty is conducted.

## 4. Application

To illustrate the developed method, an indirect fatigue test was conducted on the CSCRM. The fatigue life and its uncertainty were evaluated based on Equation (2) and the UQ method. The results showed that the fatigue life of the CSCRM could be captured using the developed method. To further verify the UQ method, Ji and Jiang’s tests [13] were used to verify the performance of the UQ method for the CSCRM. Moreover, the fatigue life of the CSCRM was characterized using the UQ method.

### 4.1. Indirect Fatigue Test

Determination of the fatigue life and its uncertainty is essential for reasonably estimating the performance of the CSCRM. In this study, the test data, generated by the indirect tensile fatigue test presented in Section 2, were used to characterize the uncertainty of the fatigue life. The fatigue lives of the CSCRMs were obtained for various combinations of the contents of the ARWMs, CRWMs, NAs, and cement. The fatigue test results are listed in Appendix B. The relationship between the fatigue life and composition content is given by Equation (2). The fatigue lives of various CSCRMs are listed in Appendix B.

To determine and quantify the uncertainty of the fatigue life, Equation (2) was adopted as the fatigue life to determine the unknown coefficients: *a*, *b*, *c*, and *d*. This study focused on determining the difference between the fatigue life *fl* and the test value *fl_t_*, treating these as Gaussian-distributed variables with a mean value *µ_d_*. This approach was used to derive a nonlinear function involving four fitting coefficients (*a*, *b*, *c*, and *d*) based on the following fatigue life equation:

(6) $$d_{fl}\sim N(\mu_{d},\sigma_{d}^{2})$$

(7) $$\mu_{d}=\sum_{i=1}^{n}\left({fl}_{i}-\frac{a\left[\frac{1+x_{2}}{2}+\frac{1-x_{2}}{2}sign(sign\left(d-x_{3}\right)-0.5)\right]}{x_{1}{\left(x_{0}+x_{1}\right)}^{2}+\left(x_{0}+x_{1}-b\right)\left(x_{0}+x_{1}-c\right)+x_{3}}\right)$$

A uniform distribution was assigned to the unknown coefficients of the fatigue equation (*a*, *b*, *c*, and *d*) due to limited information about their true values, as described below:

(8) a~U(5000,150000)

(9) b~U(0,1)

(10) c~U(−1,0)

(11) d~U(0.5,0.8)

After specifying the above model in the PyMC3 (version 3.11.5) software, the next step involved the estimation of the posterior distribution of the unknown coefficients of the fatigue life (*a*, *b*, *c*, and *d*) in Equation (2). The indirect fatigue test generated 168 sets of test data under different conditions. The 168 sets were randomly divided into two groups: 125 test data points were used to determine the fatigue equation, and the remaining 34 test data points were used to verify the UQ performance.

**Figure 2:**
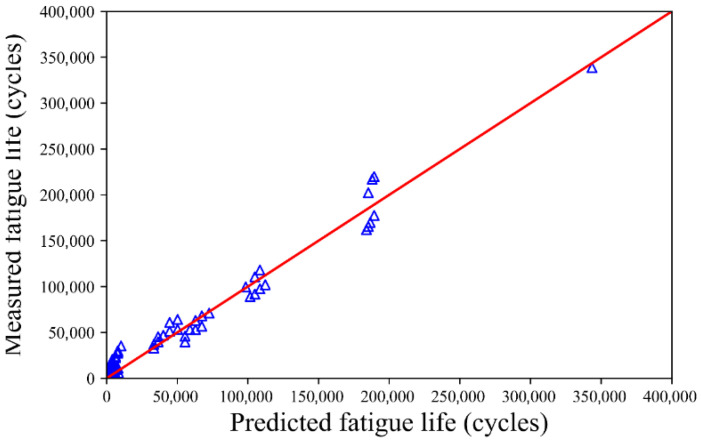
Comparison of the fatigue life between tested values and those predicted by UQ.

According to the procedure described in Section 3.3, the MCMC was used to quantify the uncertainty of the fatigue life using the aforementioned 125 groups of fatigue test data. The mean values and standard variances of the unknown coefficients of the fatigue life (*a*, *b*, *c*, and *d*) are listed in Table 1. The mean values of the unknown coefficients match well with the values obtained using Equation (2). This indicates that UQ characterizes the fatigue life of the CSCRM well. A comparison between the fatigue life of the test data and the mean value obtained by UQ is shown in Figure 2. The value of *R*^2^ is 0.9753 when calculated using Equation (2), whose unknown coefficients *a*, *b*, *c*, and *d* are determined using UQ. The mean and maximum residual between the fatigue test results and the predicted results are 1115.17 and 5888.63 cycles, respectively. It is evident that the fatigue life predicted using UQ is very close to the test fatigue life. This proves that UQ is an excellent tool for predicting the relationship between the fatigue life, material composition, and stress level.

**Figure 3:**
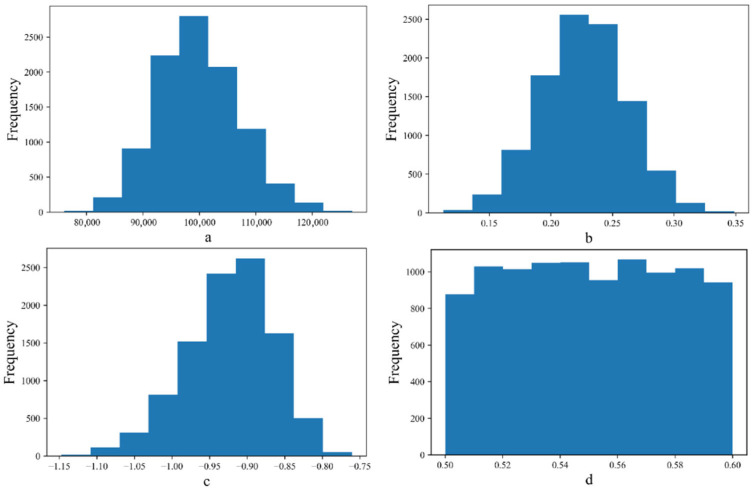
Uncertainty regarding the unknown coefficient in the fatigue life equation. (**a**) Coefficient *a* in Equation (2), (**b**) Coefficient *b* in Equation (2), (**c**) Coefficient *c* in Equation (2), (**d**) Coefficient *d* in Equation (2).

**Figure 4:**
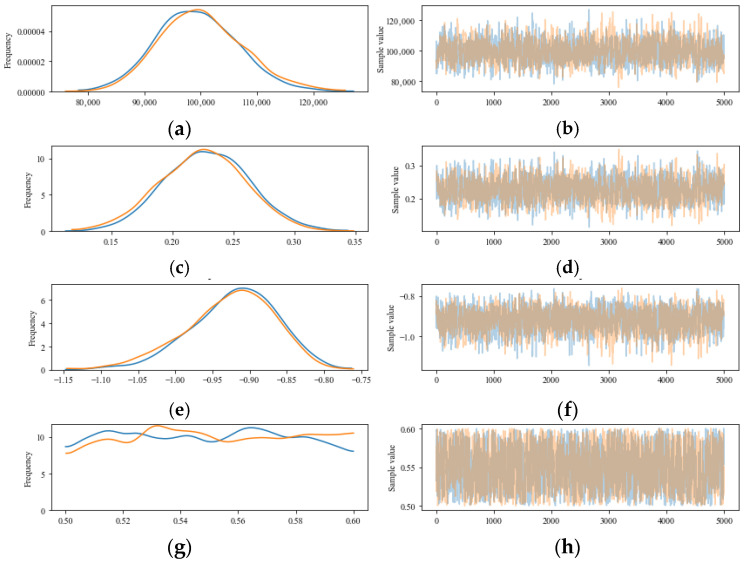
Trace distribution of samples of the unknown coefficient in fatigue life (Equation (2)) based on the MCMC. (**a**) Frequency of coefficient *a*; (**b**) Sampling value of coefficient *a*; (**c**) Frequency of coefficient *b*; (**d**) Sampling value of coefficient *b*; (**e**) Frequency of coefficient *c*; (**f**) Sampling value of coefficient *c*; (**g**) Frequency of coefficient *d*; (**h**) Sampling value of coefficient *d*.

On the one hand, uncertainty quantification (UQ) can determine the mean values of the unknown coefficients in the fatigue life equation; on the other hand, it can also evaluate the uncertainties of these coefficients based on test data. Figure 3 and Figure 4 illustrate the uncertainty characteristics of the unknown coefficients in the fatigue equation and the sample traces obtained using the MCMC, respectively. Blue and orange lines represented the results of two times MCMC simulation in Figure 4. This shows that UQ is independent of the prior distribution and can capture the uncertainty property of the fatigue behavior based on Equation (2). It proved that the posterior property of *a*, *b*, *c*, and *d* could be determined on the basis of a prior uniform distribution. It is feasible to determine the known coefficients for the fatigue equation and quantify their uncertainty based on UQ.

**Figure 5:**
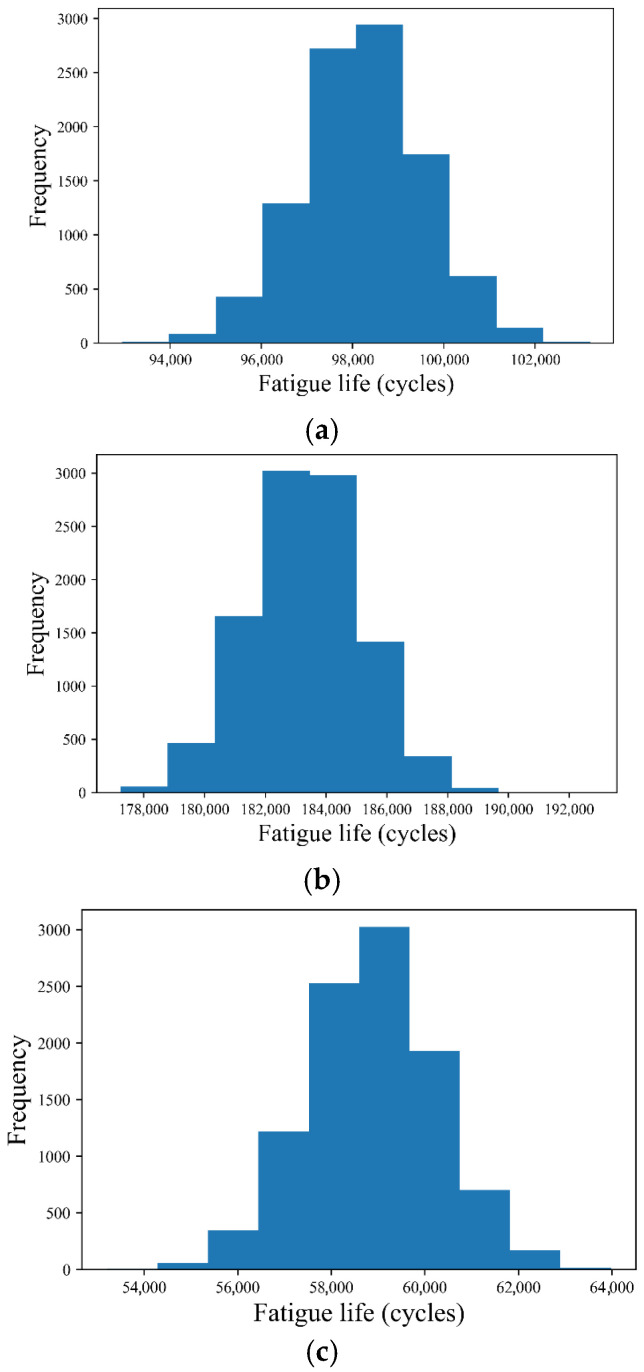
The predicted fatigue life and its uncertainty for three cases: (**a**) Case 1; (**b**) Case 2; (**c**) Case 3.

In this study, the above-mentioned 34 test data points were used to verify the performance of UQ. The fatigue life was estimated using uncertainty from UQ for the other three CSCRMs. The value of *R*^2^ was 0.9666 when calculated using Equation (2), whose unknown coefficients *a*, *b*, *c*, and *d* were determined using UQ. Figure 5 illustrates the distribution of fatigue life for the three cases (Case 1, Case 2, and Case 3) based on the 34 data points. The fatigue lives obtained from the test are 83,347, 198,483, and 45,588 cycles, respectively.

### 4.2. Ji and Jiang’s Test

To further demonstrate and verify the developed method, UQ is used to evaluate the fatigue life and its uncertainty based on Ji and Jiang’s fatigue test data (Appendix C) [13]. Four samples are successfully tested for each stress level and CSCRM composition. The results presented in Table A6 are the mean values of the four samples. The relationship between the material composition and the fatigue life is given by Equation (2). The fatigue lives of Ji and Jiang’s tests are listed in Appendix C. Test data can be divided into two groups. The test data for a cement content of 4% are selected to determine the fatigue equation, and the others are used to verify the performance of UQ.

In this section, the fatigue life *fl* (Equation (5)) and the mean of the difference a mean value *µ_d_* (Equation (6)) are the same as those obtained in Section 4.1. A uniform distribution is used to determine the unknown coefficients *a*, *b*, *c*, and *d* of the fatigue equation based on weak information regarding their actual values:

(12) a~U(5000,150000)

(13) b~U(0,1)

(14) c~U(−1,0)

(15) d~U(0.5,0.8)

**Figure 6:**
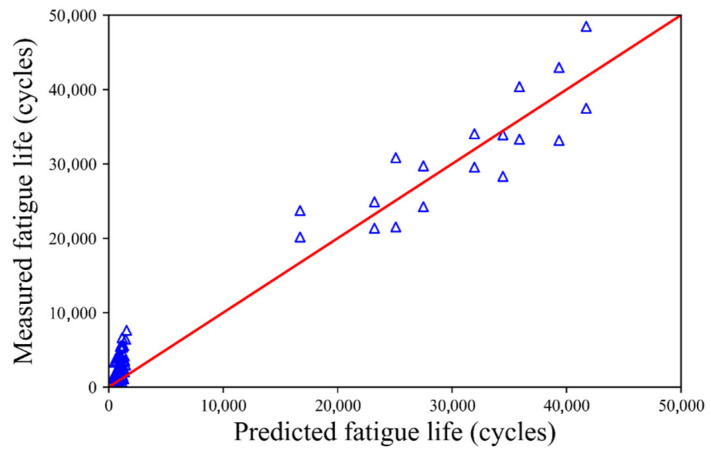
Comparison of the fatigue life values from the test and those predicted using UQ for Ji and Jiang’s test.

The mean values and standard deviations of the unknown coefficients for fatigue life *a*, *b*, *c*, and *d* are listed in Table 2. The mean values of these coefficients align closely with the results obtained from Equation (2). This also shows that UQ characterizes the fatigue life of the CSCRM well. A comparison of the fatigue life between the test data and the mean value obtained using UQ is shown in Figure 6. The value of *R*^2^ is 0.9810 using Equation (2), whose unknown coefficients *a*, *b*, *c*, and *d* are determined using the UQ. It is evident that the fatigue life predicted by using UQ is very close to the test fatigue life. This also proves that UQ can be used to evaluate the fatigue life based on the material composition and stress level, indicating that UQ possesses an acceptable prediction ability. 

**Figure 7:**
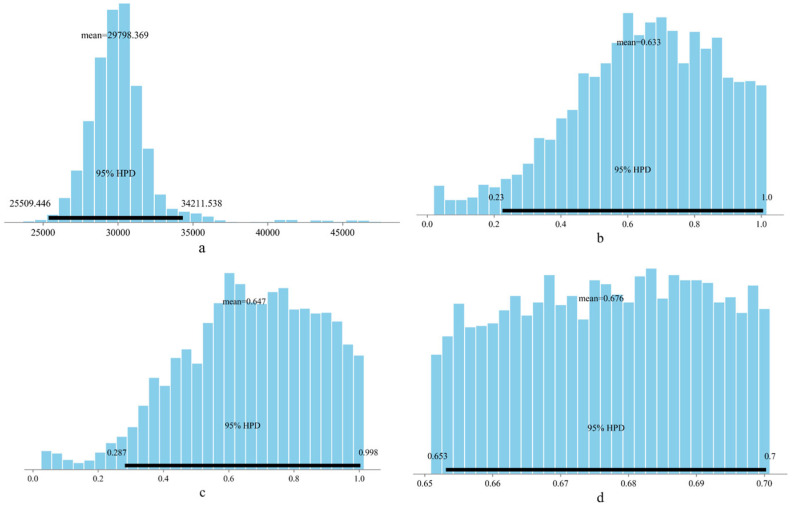
Uncertainty of the unknown coefficient in the fatigue life equation (**a**) Coefficient *a* in Equation (2); (**b**) Coefficient *b* in Equation (2); (**c**) Coefficient *c* in Equation (2); (**d**) Coefficient *d* in Equation (2).

**Figure 8:**
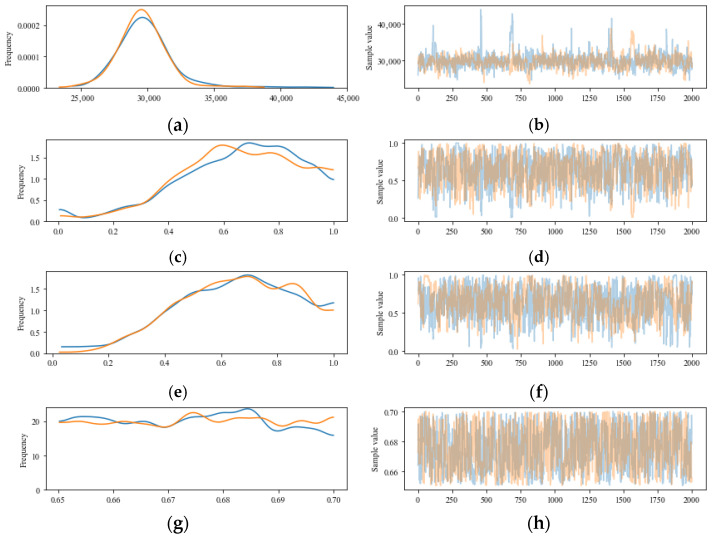
Trace distribution of samples of the unknown coefficient in fatigue life (Equation (2)) based on the MCMC for Ji and Jiang’s Test. (**a**) Frequency of coefficient *a*; (**b**) Sampling value of coefficient *a*; (**c**) Frequency of coefficient *b*; (**d**) Sampling value of coefficient *b*; (**e**) Frequency of coefficient *c*; (**f**) Sampling value of coefficient *c*; (**g**) Frequency of coefficient *d*; (**h**) Sampling value of coefficient *d*.

Figure 7 illustrates the uncertainty associated with the unknown coefficients in the fatigue equation, along with the 95% highest posterior density (HPD). In this figure, the posterior distributions for coefficients *a*, *b*, and *c* closely resemble a normal distribution, while coefficient d exhibits an approximately uniform distribution, reflecting the prior uniform distribution. The 95% HPD shown in Figure 7 further confirms that uncertainty quantification (UQ) is independent of the prior distribution and effectively captures the uncertainty associated with fatigue behavior, as described by Equation (2). Additionally, the unknown coefficients derived through UQ encompass the values obtained from Equation (2) using symbolic regression [31]. Figure 8 displays the traces of the samples calculated using Markov Chain Monte Carlo (MCMC) methods (blue and orange lines represented the results of two times MCMC simulation), reinforcing the conclusion that the posterior properties of coefficients *a*, *b*, *c*, and *d* can be established based on a prior uniform distribution. Overall, UQ serves as a reliable approach for determining the known coefficients of the fatigue equation and quantifying their associated uncertainties.

**Figure 9:**
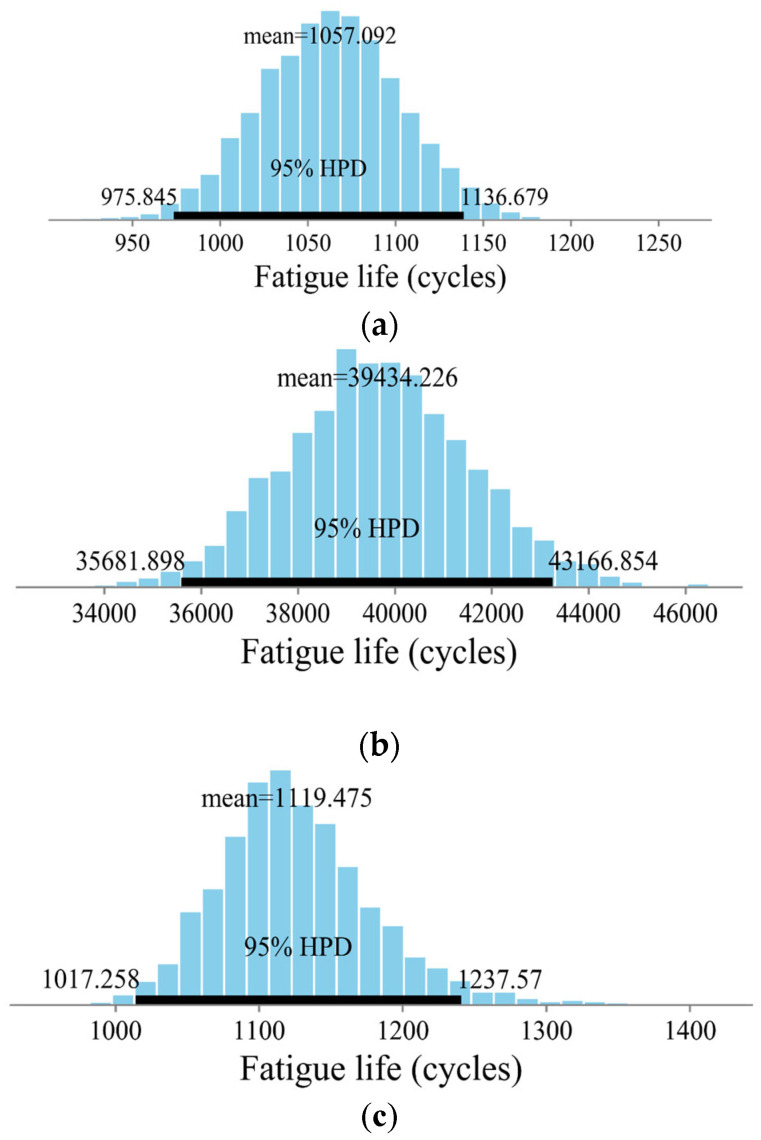
The predicted fatigue life and its uncertainty for the three cases based on Ji and Jiang’s Test: (**a**) Case 1; (**b**) Case 2; (**c**) Case 3.

Uncertainty and random errors are unavoidable in the CSCRMs owing to the complexity of the fatigue behavior and the material environment. The traditional fatigue equation neglects uncertainty and error to obtain a deterministic fatigue life, which is inconsistent with the practical behavior of the material. UQ can be used to obtain the mean fatigue life and determine the uncertainty of the fatigue life. Once the uncertainty of the unknown coefficient in the fatigue equation is determined, an uncertainty analysis was conducted using the MCMC. The fatigue life was also estimated based on the uncertainty obtained using UQ for the other three CSCRMs—Case 1, Case 2, and Case 3. Figure 9 shows the fatigue life, mean value, and 95% confidence interval of the three cases. The mean fatigue lives are 1057.092, 39,434.226, and 1119.475 cycles, respectively. The fatigue lives obtained from the tests are 1187, 42,966, and 1103 cycles, and the relative errors are 10.95, 8.00, and 1.62%, respectively.

The developed UQ framework enables the analysis and computation of the designed fatigue life by examining the uncertainties present in experimental data and fatigue life equations. In this study, a large number of experiments were performed, and the composition of each component in the cement-stabilized cold recycled mixture (CSCRM) was categorized with greater precision. This approach allows the research framework to consider a wider range of possibilities, thereby improving the prediction of the CSCRM′s fatigue life. A detailed analysis of the experimental results is provided below:

- Because the bond between the natural aggregate (NA) and cement is stronger than the bond between the recycled aggregate (RA) and cement, increasing the amount of the NA can prolong the fatigue life of the CSCRM. In contrast, a higher proportion of RAs—consisting of ARWMs and CRWMs—negatively affects the fatigue life extension of the CSCRM. This study also shows that the RA has a more pronounced effect on the CSCRM′s fatigue life when present in low amounts. Additionally, applying a high level of stress diminishes the impact of a low RA content on the mixture′s fatigue life and decreases the mixture′s fatigue life sensitivity to variations in the low RA content.
- As cement is a binder, an increase in the cement content exhibits a positive correlation with the fatigue life of the CSCRM; however, the sensitivity of the CSCRM′s fatigue life to the cement content is lower than for the RA.
- When the NA content remains constant, the asphalt coating on the surface of ARWMs obstructs water from reaching the aggregate surface, which subsequently influences the interaction between the aggregate and cement, negatively affecting the fatigue life of the CSCRM. In contrast, increasing the amount of the CRWM enhances the fatigue life of the CSCRM.
- The stress level has an inverse impact on fatigue life; however, similar to the RA content, its effect on the fatigue life diminishes as the stress level rises. Under low stress conditions, the RA content and the amount of CRWM in cement-stabilized cold recycled mixtures (CSCRMs) need to be carefully controlled.

The laboratory test data from this study reveal the influence of the law of different recycled aggregate contents on the fatigue life of a cement-stabilized recycled asphalt mixture (CSCRM) under various load conditions. Compared with previous studies, this study further analyzes the effects of different components on the fatigue life of a CSCRM, especially the influence of the composition ratio of CRWMs and ARWMs. In addition, based on the fatigue life equation, this study establishes a CSCRM fatigue life prediction framework that accounts for uncertainties. This framework not only details the information of each component of CSCRM but also integrates the influencing factors of different stress levels, fully considering the uncertain factors affecting fatigue life by using probabilistic methods. Meanwhile, further verification is conducted by adopting the test data of Ji and Jiang, which confirms the practicality and effectiveness of this prediction framework.

In addition, the proposed approach can be adopted for other civil materials, rather than only for CSCRMs. For other civil materials, once experimental fatigue data in the case of different conditions (e.g., materials compositions, stress level, etc.) and the corresponding fatigue equation are obtained, the experimental data of fatigue life, the condition factors (e.g., material compositions, stress level, etc.), and the coefficients of the fatigue equation can be input into the proposed approach. The predicted fatigue life and the uncertainty parameters of the coefficients can be automatically output by the proposed approach.

## 5. Conclusions

Determining the fatigue life of pavement materials is critical to the design, analysis, construction, and operation of pavement engineering. Fatigue tests are a commonly used method for characterizing the fatigue properties of pavement materials. However, fatigue test data are scattered, random, and uncertain due to the materials′ complexity. Characterizing reasonable uncertainty during fatigue testing is essential to predicting and determining fatigue life scientifically. The traditional empirical formula does not address the aforementioned uncertainty. This study developed a UQ framework to deal with uncertainty when determining the fatigue life based on an empirical formula using probabilistic programming. The unknown coefficient of the empirical formula was quantified and determined based on test data and PyMC3. The developed method was illustrated and verified using cement-stabilized cold recycled mixtures based on an indirect fatigue test. The results of this study can be summarized as follows:

- The developed method scientifically considered the uncertainty during fatigue testing by combining probabilistic programming, fatigue test data, and an empirical formula. Probabilistic programming was employed to characterize the fatigue behavior and quantify the associated uncertainty during fatigue testing. The developed method provides a scientific, feasible, and helpful way for dealing with the uncertainty regarding the fatigue life of pavement materials.
- The developed method was illustrated and verified based on an empirical formula by two independent CSCRM datasets. PyMC3 is a reliable probabilistic programming package for quantifying uncertainty during the fatigue test.
- The developed method quantified the uncertainty of fatigue life by determining the unknown coefficient of the empirical formula. The empirical formula characterizes the fatigue behavior, and its selection is critical to the developed method. More empirical formulas should be verified and investigated in future studies.
- The developed method was applied to a CSCRM and exhibits excellent performance for the quantification of uncertainty, avoiding complex statistical computations. It should be further applied to other civil materials in future studies.

## Figures and Tables

**Table 1:** The value of the unknown coefficient in the fatigue equation and its associated uncertainty.

Unknown Coefficient	UQ		Equation (2)	Relative Error (%)
	Mean Value	Standard Deviation		
*A*	98,680.28	6029.57	100,000	1.32
*B*	0.23	0.030	0.2	15.96
*C*	−0.91	0.046	−1	8.71
*D*	0.55	0.028	0.562	1.73

**Table 2:** The value of the unknown coefficient in the fatigue equation and its uncertainty.

Unknown Coefficient	UQ		Equation (2)	Relative Error (%)
	Mean Value	Standard Deviation		
*a*	29,798.369	2525.195	33,207.78	10.49
*b*	0.633	0.2173	0.66	4.00
*c*	0.647	0.2136	0.66	2.64
*d*	0.676	0.0143	0.70	3.60

**Figure A1:**
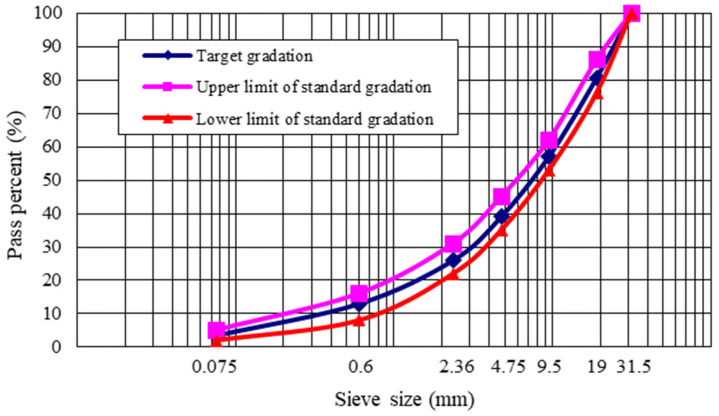
Aggregate gradation.

**Figure A2:**
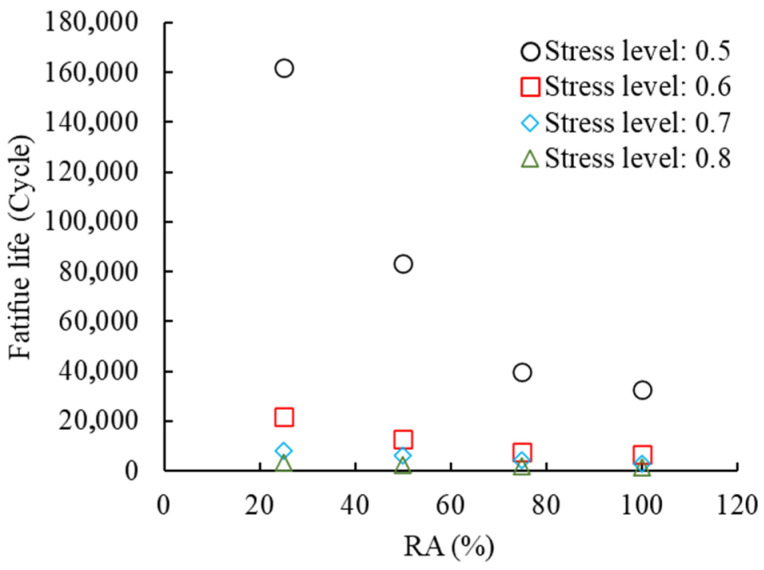
Effect of the RA and stress level on fatigue life.

**Figure A3:**
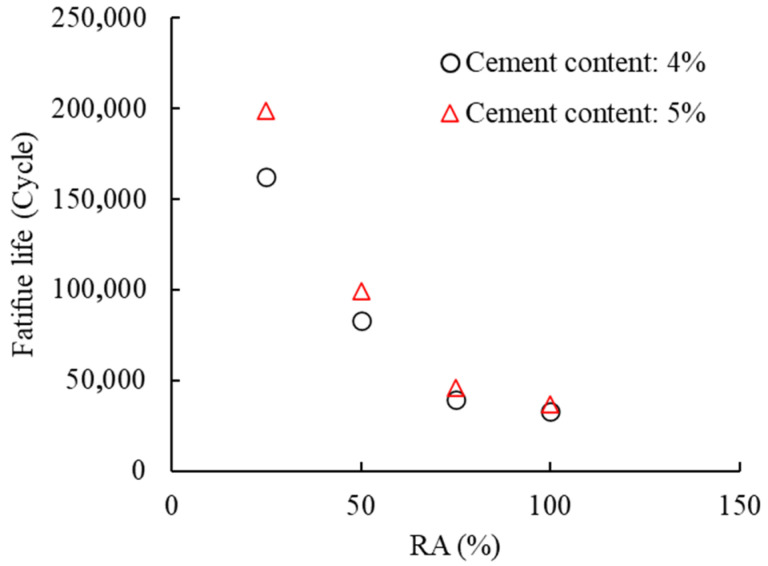
Effect of the RA and cement content on fatigue life.

**Figure A4:**
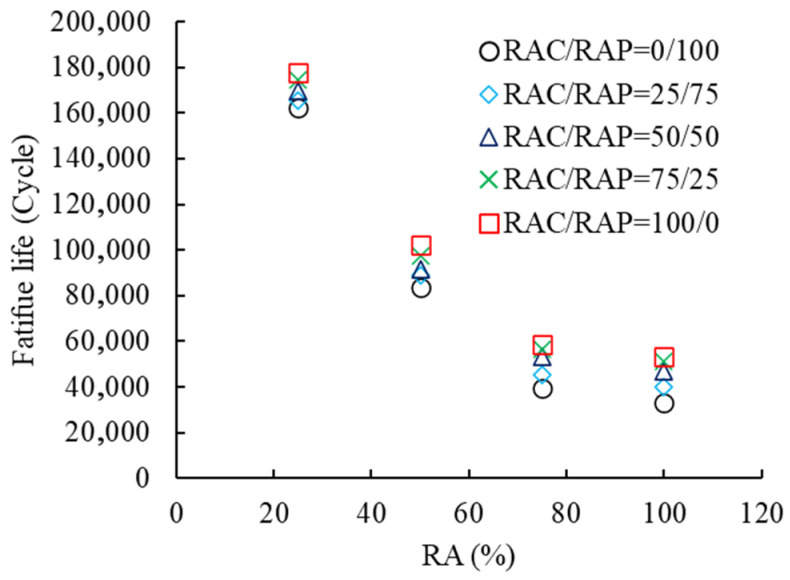
Effect of the RA and ARWM /RAP on fatigue life.

## Data Availability

The original contributions presented in this study are included in the article. Further inquiries can be directed to the corresponding author.

## Appendix A. Main Characteristics of Raw Materials and CSCRMs

**Table A1:** Technical properties of cement.

Index		Result
CaO content (%)		66.7
SiO_2_ content (%)		21.9
Al_2_O_3_ content (%)		6.1
Fe_2_O_3_ content (%)		4.6
SO_3_ content (%)		2.2
MgO content (%)		4.0
Cl-content (%)		0.037
Fineness (%)		1.1
Setting time (min)	Initial setting	196
	Final coagulation	277
Stability	Le chatelier soundness	Qualified
Flexural strength (MPa)	3-day	4.8
	28-day	8.2
Compressive strength (MPa)	3-day	23.9
	28-day	48.1

**Table A2:** Technical properties of the NA.

Material Specification	Apparent Density (g·cm^−3^)	Water Absorption (%)	Needle Sheet Content (%)	Natural Water Content (%)	Crushing Value (%)
19~37.5 mm	2.612	1.06	10.1	0.2	11.6
9.5~19 mm	2.736	1.10	10.7		
4.75~9.5 mm	2.719	1.29	11.2		
0~4.75 mm	2.377	1.67	—		

**Table A3:** Technical properties of the RA.

Material	Apparent Density (g·cm^−3^)	Natural Water Content (%)	Water Absorption (%)	Needle Sheet Content (%)	Crushing Value (%)
ARWM	2.496	4.8	7.12	4.1	13.7
CRWM	2.404	6.1	9.81	5.5	15.9

**Table A4:** The optimum water content and the maximum dry density of the CSCRM.

Cement Content (%)	NA Content (%)	ARWM Content (%)	CRWM Content (%)	OMC (%)	MDD (g·cm^−3^)	Splitting Strength (MPa)	
						Mean	Std.
4	100	0	0	4.5	2.389	1.74	0.162
5	100	0	0	4.6	2.401	2.29	0.039
4	75	0	25	4.6	2.311	1.18	0.017
	50	0	50	4.7	2.216	0.92	0.089
	25	0	75	4.9	2.136	0.78	0.056
	0	0	100	5.0	2.103	0.69	0.021
5	75	0	25	4.7	2.322	1.87	0.180
	50	0	50	4.8	2.224	1.55	0.128
	25	0	75	4.9	2.144	1.28	0.065
	0	0	100	5.0	2.111	1.11	0.082
4	75	6.25	18.75	5.0	2.317	1.21	0.022
	50	12.5	37.5	5.3	2.243	0.95	0.083
	25	18.75	56.25	5.6	2.164	0.81	0.041
	0	25	75	5.9	2.105	0.73	0.031
5	75	6.25	18.75	5.1	2.332	1.90	0.099
	50	12.5	37.5	5.4	2.258	1.60	0.043
	25	18.75	56.25	5.7	2.177	1.33	0.008
	0	25	75	6.0	2.113	1.16	0.049
4	75	12.5	12.5	5.3	2.328	1.23	0.059
	50	25	25	5.8	2.276	0.98	0.071
	25	37.5	37.5	6.4	2.210	0.84	0.063
	0	50	50	6.9	2.105	0.77	0.048
5	75	12.5	12.5	5.4	2.346	1.94	0.028
	50	25	25	5.9	2.292	1.68	0.003
	25	37.5	37.5	6.6	2.221	1.43	0.095
	0	50	50	6.9	2.114	1.21	0.075
4	75	18.75	6.25	5.8	2.345	1.28	0.075
	50	37.5	12.5	6.6	2.301	1.02	0.080
	25	56.25	18.75	7.4	2.241	0.86	0.023
	0	75	25	8.2	2.107	0.80	0.037
5	75	18.75	6.25	5.9	2.358	2.03	0.188
	50	37.5	12.5	6.5	2.312	1.76	0.065
	25	56.25	18.75	7.5	2.250	1.51	0.127
	0	75	25	8.3	2.115	1.34	0.039
4	75	25	0	6.7	2.358	1.35	0.047
	50	50	0	7.6	2.321	1.05	0.041
	25	75	0	8.6	2.265	0.89	0.075
	0	100	0	9.5	2.109	0.83	0.009
5	75	25	0	6.6	2.368	2.15	0.029
	50	50	0	7.7	2.331	1.88	0.181
	25	75	0	8.7	2.270	1.60	0.085
	0	100	0	9.6	2.116	1.50	0.035

## Appendix B. The Results of the Indirect Fatigue Test

**Table A5:** Fatigue test results.

ARWM Content (%)	CRWM Content (%)	NA Content (%)	Cement Content (%)	Fatigue Life Under Different Stress Levels				Correlation Coefficient
				0.5	0.6	0.7	0.8	
0	0	100	4	279,492	35,196	10,986	4891	0.9553
			5	338,716	43,938	13,691	6133	0.9564
0	100	0	4	32,908	6633	3187	1338	0.9666
0	75	25		39,685	7821	4537	1821	0.9539
0	50	50		83,347	12,881	6172	2612	0.949
0	25	75		162,048	21,697	8001	3216	0.9593
0	100	0	5	36,969	7481	3555	1498	0.9672
0	75	25		45,935	9101	5198	2111	0.9549
0	50	50		99,600	15,484	7376	3089	0.9506
0	25	75		198,483	26,129	9811	3769	0.9607
25	75	0	4	39,780	7710	4061	1481	0.9662
18.75	56.25	25		45,588	8991	5088	1987	0.9579
12.5	37.5	50		89,032	13,413	6603	2762	0.9463
6.25	18.75	75		165,524	22,266	8399	3361	0.9588
25	75	0	5	45,318	8766	4589	1671	0.9666
18.75	56.25	25		53,223	9999	5943	2327	0.9501
12.5	37.5	50		106,393	16,079	7719	3300	0.9466
6.25	18.75	75		202,326	27,229	10,267	4176	0.9577
50	50	0	4	46,861	8980	4585	1621	0.9691
37.5	37.5	25		53,164	9788	5555	2211	0.9519
25	25	50		91,826	14,387	7461	2883	0.9509
12.5	12.5	75		169,841	22,986	8829	3482	0.9593
50	50	0	5	54,379	10,417	5318	1878	0.9692
37.5	37.5	25		63,132	11,581	6596	2629	0.9512
25	25	50		110,650	17,633	9001	3474	0.9532
12.5	12.5	75		208,769	28,267	10,837	4226	0.9602
75	25	0	4	51,413	9555	5715	2092	0.953
56.25	18.75	25		56,707	10,339	6711	2555	0.9408
37.5	12.5	50		97,516	18,011	8130	3316	0.9667
18.75	6.25	75		174,718	23,461	9317	3938	0.9521
75	25	0	5	60,861	11,316	6699	2468	0.9536
56.25	18.75	25		68,111	12,138	8111	3071	0.9362
37.5	12.5	50		117,981	19,962	9878	3939	0.957
18.75	6.25	75		216,978	29,068	11,400	4799	0.9529
100	0	0	4	53,379	9869	6496	2266	0.9455
75	0	25		58,551	10,677	7419	2779	0.9337
50	0	50		102,044	20,476	8596	3687	0.9718
25	0	75		177,584	23,976	10,007	4139	0.9513
100	0	0	5	64,134	12,117	7991	2716	0.948
75	0	25		70,993	13,131	9099	3361	0.936
50	0	50		125,003	21,871	10,981	4531	0.9557
25	0	75		219,886	30,001	12,388	5119	0.9526

## Appendix C. Ji and Jiang’s Test Data

**Table A6:** Ji and Jiang’s test results.

ARWM Content (%)	CRWM Content (%)	NA Content (%)	Cement Content (%)	Fatigue Life Under Different Stress Levels					Correlation Coefficient
				0.85	0.80	0.75	0.70	0.65	
30	70	0	3	405	1150	1635	3353	20,166	0.9334
24	56	20		605	1403	1914	3536	21,517	0.9088
18	42	40		755	1421	2238	4068	28,318	0.8976
25	75	0	4	534	1231	1919	3917	23,720	0.9312
18.75	56.25	25		659	1383	2579	4170	30,834	0.9157
6.25	18.75	75		1103	2041	2956	5561	33,910	0.8975
50	50	0	3	640	1258	1929	3965	21,349	0.9266
40	40	20		988	1762	2865	4585	29,555	0.8941
30	30	40		1147	1961	2878	5554	33,166	0.8938
50	50	0	4	724	1331	2210	4628	24,892	0.9307
40	40	20		1187	2084	3293	5347	34,047	0.8903
30	30	40		1458	2470	3550	6424	42,966	0.8745
100	0	0	3	822	1449	2324	3923	24,235	0.8988
80	0	20		1551	2033	3566	5471	33,319	0.8655
60	0	40		1470	2474	4119	6623	37,477	0.9036
100	0	0	4	803	1231	1919	3917	29,732	0.8714
80	0	20		659	1383	2579	4170	40,381	0.8947
60	0	40		1103	2041	2956	7622	48,482	0.9082
